# Comparing the pregnancy outcomes of cleavage and blastocyst stage in frozen embryo transfer cycles: A cross-sectional study

**DOI:** 10.18502/ijrm.v21i11.14656

**Published:** 2023-12-19

**Authors:** Zahra Parsafar, Razieh Dehghani-Firouzabadi

**Affiliations:** ^1^Department of Obstetrics and Gynecology, Shahid Sadoughi University of Medical Sciences, Yazd, Iran.; ^2^Research and Clinical Center for Infertility, Yazd Reproductive Sciences Institute, Shahid Sadoughi University of Medical Sciences, Yazd, Iran.

**Keywords:** Blastocyst, Ovum cleavage stage, Embryo transfer.

## Abstract

**Background:** In vitro fertilization has advanced in many ways, and new techniques are challenging. Blastocyst transfer is an alternative method for embryo transfer (ET) to improve in vitro fertilization outcomes. **Objective:** The present study was performed to determine the effect of pregnancies resulting from ET in the blastocyst stage compared to the cleavage stage in frozen cycles to select a better method of assisted reproduction. **Materials and Methods:** This cross-sectional study was conducted on 194 women who referred to the Yazd Reproductive Sciences Institute, Yazd, Iran, between April 2019 and December 2020. They had a frozen ET as either cleavage or blastocyst (n = 97/each group). The study compared the pregnancy and fetal outcomes in the 2 groups of ET at the cleavage and blastocyst stages. **Results:** The results showed that the blastocyst stage group had higher levels of anti-Mullerian hormone, ovule number, 2 pronuclear number, and embryo number than the cleavage stage group. The frequency of chemical pregnancies was 52.6% and 36.1% in blastocyst and cleavage group respectively (p = 0.02). Also, the frequency of clinical pregnancies was 41.2% and 22.7% in blastocyst and cleavage group respectively (p < 0.001). No statistically significant difference was observed between 2 groups in abortion, preterm delivery, multiple births, preterm premature rupture of membranes, gestational diabetes and preeclampsia, ectopic pregnancy, neonatal hospitalization in Neonatal Intensive Care Unit, and fetal abnormalities (p > 0.05). **Conclusion:** The results showed that transmission in the blastocyst stage compared to the cleavage stage is associated with an increase in chemical and clinical pregnancy, while other pregnancy outcomes are the same in both groups.

## 1. Introduction

Assisted reproductive technologies (ART) include all strategies used to manipulate oocytes outside the body (1). There have been many improvements in the ATRs over the last 3 decades; introducing the freezing techniques can be named as one of them evolving alongside the ATRs (2). The reduced risk of multiple pregnancies with less fresh embryo transfer (ET) is probably one of the most important reasons for the development of the freezing methods (3). Since multiple pregnancies are one of the major risk factors for miscarriage and premature birth, we do not have to transfer all the fresh embryos using this method, and the rest can be frozen for the future (4-6). Another reason for freezing is the insufficiency of the uterine endometrium in the ultrasound performed before the transfer of a new fetus (7). The results of studies done in fresh ET vs. frozen embryos are controversial. Many have shown that the rate of fertility outcome and pregnancy is higher when using new fetuses (8-10); however, others claim that no significant difference was observed between the outcomes of the mentioned methods (11). A meta-analysis done in 2012 expressed that the rate of fetal replacement and occurrence of clinical pregnancy was significantly higher when using frozen embryos (5).

The possibility of fertility after thawing the egg directly depends on the egg's quality and its maturation (12). The embryo quality plays an important role in the success rate of the transfer; in such a way that in embryos with at least half of healthy cells after thawing, the survival rate is 50-65%. Furthermore, some studies believe that the abortion rate is higher in a 3-day-old fetus (13, 14). Implantation occurs 5-7 days after fertilization in a normal pregnancy, while in a normal in vitro fertilization (IVF), the 2-3 days old fetus is transferred to the uterus during the cleavage stage. The new technology provides the situation for the fetus so that it can reach the blastocyst stage (6-5 days old fetus) and then be transferred to the uterus. The blastocyst transfer has increased the delivery rate in the new cycles (15, 16). A couple of investigations have reported a higher rate of delivery followed by the blastocyst transfer than the cleavage stage (15, 17). A study presented that the rate of pregnancy was higher in the blastocyst transfer than the cleavage phase in the fresh cycles, yet the results were opposite for the frozen embryos, and as a result, the overall rate of pregnancy in the frozen and fresh cycles was the same (18).

The current study aimed to evaluate the outcome of pregnancies resulting from ET in the blastocyst stage vs. the cleavage stage in frozen cycles to make a better decision and choose the best treatment option.

## 2. Materials and Methods

This cross-sectional study was conducted on 194 women who referred to Reproductive Sciences Institute, Yazd, Iran from April 2019-December 2020, and had done ET in the blastocyst and cleavage stage were included. Women aged between 18 and 45 yr with a history of uterine abnormalities, chronic diseases such as systemic lupus erythematosus, diabetes mellitus, hypertension, liver, kidney, heart diseases, smokers, drugs and alcohol users, cases with egg donation and surrogacy pregnancies, severe azoospermia, and endometriosis were excluded from this study. All women had previously undergone IVF or intracytoplasmic sperm injection with embryo freezing and had failed were enrolled. Participants were divided into 2 groups (cleavage and blastocyst stages) based on the age of the fetus during transfer. Pregnancy outcomes including preterm delivery, premature rupture of membranes, pre-eclampsia, gestational diabetes, miscarriage, ectopic pregnancy, cesarean rate, and fetal consequences including multiple births, fetal abnormalities, birth weight, need for neonatal intensive care unit (NICU), intrauterine fetal death, and growth restrictions were compared in both groups. Data related to the pregnancy outcomes were collected using the stored medical records and filling out a researcher-made questionnaire. The questionnaire included questions about the maternal demographic information, duration of infertility, cause of infertility, history of abortion, gestational age, gestational diabetes or hypertension, need for a cesarean, preterm delivery, condition of membranes, fetal weight, need for NICU hospitalization, fetal abnormalities, and intrauterine death.

Day 2 after retrieval of oocytes, embryos were assessed. After 2-stage loading with the comparison solution containing methyl sulfoxide and ethylene glycol and a glass freezing solution containing methyl sulfoxide, ethyl glycol, and 0.5 mol/L sucrose, and then by using a thin capillary tube glass was loaded in cryotron. Only a thin layer covering the embryo was left before immersing the samples in liquid nitrogen.

Samples were thawed after 2 months of freezing. The straws were placed in the environment for 30 sec and then immersed in 30 C water for 30 sec. Thawed embryos survived morphologically with 50% or more blastomeric intact and no evidence of damage to the zona pellucida. All embryos of the blastocyst group were transferred to serial media until blastocyst development. In the cutting stage group, the embryos were cultured only for 1 day. Only those blastocysts were used which had a large blastocoel (at least half the size of the embryo), the internal cell mass could be recognized, and whose trophectoderm was formed.

To prepare the endometrium in both groups, estradiol valerate (estradiol valerate, Aburaihan CO, Tehran, Iran) was used orally at one dose of 6 mg per day from the day 2 of the menstrual cycle. In order to evaluate the thickness of the endometrium, we began doing vaginal ultrasonography from the $13^{\text{th}}$ day of the menstrual cycle. When the thickness of the endometrium reached > 8 mm, 100 mg of progesterone (progesterone, Aburihan Co., Tehran, Iran) was injected daily. Administration of estradiol and progesterone was continued until fetal heart activity was observed using ultrasound. Embryo thawing was done in both groups 2 days after the start of progesterone injection. Embryos of cut group and blastocyst were transferred 1 and 3 days after thawing, respectively.

Serum beta human chorionic gonadotropin ≥ 50 IU/L was described as positive chemical pregnancy. We checked beta human chorionic gonadotropin 10-12 days after ET in cleavage and blastocyst group, respectively.

### Sample size

The significance level and the test power were considered 5% and 80%, respectively. A number of 93 cases were included in each group based on clinical pregnancy results; 30% in the cleavage method and 50% in the blastocyst method.


$$\frac{{\left(2/c+2\right)}^{2}{[}_{1}\left(1{-}_{1}\right){+}_{2}\left(1{-}_{2}\right)]}{{{[}_{1}{-}_{2}]}^{2}}$$

### Ethical considerations

Oral consent was obtained from women. This study was approved by the Ethics Committee of Shahid Sadoughi University of Medical Sciences, Yazd, Iran (Code: IR.SSU.MEDICINE.REC.1399.153).

### Statistical analysis

All registered data were analyzed using SPSS software version 20 for windows (SPSS, Chicago, IL). The mean ± SD index was used for quantitative variables with normal distribution. The Chi-square test and Students *t* test were used to compare data between the 2 groups. P-values < 0.05 were considered significant for all analyses.

## 3. Results

A total of 194 women were divided into 2 equal groups of cleavage and blastocyst transfer. Results showed that the mean of anti-Müllerian hormone, number of eggs, 2pn, and embryos were significantly higher in the blastocyst group compared to the cleavage group. However, no significant difference was found between the number of transferred embryos in groups (p = 0.42). Results showed that the causes of infertility frequency were different in the blastocyst and cleavage transfer (Table I). The frequency distribution of chemical pregnancies (positive BHCG test of blood or urine) and clinical pregnancy (fetal heart rate monitoring) in both groups showed that the frequency of both pregnancies was significantly higher in the blastocyst group. The possibility of chemical and clinical pregnancy was 1.96 times (1.1-3.49 = 95% CI) and 2.39 times (1.28-4.46 = 95% CI) higher in the blastocyst transfer, respectively.

A significant difference was observed in pregnancy outcomes such as abortion, ectopic pregnancy, multiple births, preterm birth, fetal malformations, preterm premature rupture of membranes, NICU hospitalization, type of delivery, gestational diabetes, gestational hypertension, and type of infertility in both study groups (Table II).

**Table 1:** Demographic and baseline characteristics of participants (n = 97/each)


**Variables**		**Blastocyst group**	**Cleavage group**	**P-value**
Age* (yr)		32.2 ± 4.8	32.18 ± 5.5	0.9
AMH* (ng/ml)		7.18 ± 5.29	5.1 ± 4.19	< 0.001
Cycle duration* (day)		13.02 ± 1.9	13.06 ± 1.6	0.98
Number of ovules*		23.3 ± 10.7	15.86 ± 10.12	< 0.001
Number of 2 pronuclear*		14.66 ± 8.23	9.21 ± 7.22	< 0.001
Number of embryo*		13.01 ± 7.28	7.21 ± 4.07	< 0.001
Causes of infertility**				
	**Ovulation disorder**	3.(3.1)	0 (0)	
	**Male causes**	13 (13.4)	17 (17.5)	
	**Endometriosis**	1 (1)	1 (1)	
	**Unknown**	10 (10.4)	13 (13.4)	
	**Anatomical disorder**	1 (1)	5 (5.2)	
	**Decreased ovarian reserve**	0 (0)	1 (1)	
	**Mixed**	27 (27.8)	32 (33)	
	**PCOS**	38 (39.2)	18 (18.6)	
	**Abortion**	4 (4.1)	1 (1)	
	**Ovarian failure**	0 (0)	9 (9.3)	< 0.001
*Data presented as Mean ± SD. Students' *t *test, **Data presented as n (%). Chi-square test. AMH: Anti-Müllerin hormone, PCOS: Polycystic ovary syndrome				

**Table 2:** Comparison of study outcomes in blastocyst and cleavage groups (n = 97/each)


**Outcomes**		**Blastocyst group**	**Cleavage group**	**P-value**
**Chemical pregnancy **		51 (52.6)	35 (36.1)	0.02
**Clinical pregnancy**		40 (41.2)	22 (22.7)	< 0.001
**Abortion**		7 (7.2)	2 (2.1)	0.08
**Ectopic pregnancy**		1 (1)	0 (0)	0.31
**Multiplication**		10 (10.3)	7 (7.2)	0.44
**Preterm delivery**		9 (9.3)	7 (7.2)	0.60
**Fetal abnormalities**		3 (3.1)	0 (0)	0.08
**PPROM**		1 (1)	0 (0)	0.31
**Hospitalized in NICU**		1 (1)	2 (2.1)	0.55
**Type of delivery**				
	**NVD**	1 (1)	3 (3.1)	
	**Cesarean**	96 (99)	94 (96.9)	0.30
**GDM**		4 (4.1)	1 (1)		0.17
**Hypertension**		3 (3.1)	3 (3.1)	0.99
**Infertility**				
	**Primary**	65 (67)	71 (73.2)	
	**Secondary**	32 (33)	26 (26.8)	0.34
Data presented as n (%). Chi-square test. PPROM: Preterm premature rupture of the membranes, NICU: Neonatal intensive care unit, NVD: Normal vaginal delivery, GDM: Gestational diabetes mellitus				

## 4. Discussion 

Today, paying attention to assisted reproductive methods has been effective in improving the quality of life of families dealing with infertility. But despite the remarkable progress in the science of assisted reproduction, the success rate is still not significant (19).

In women treated with ART, 2 methods of ET are considered, transfer in blastocyst stage in contrast to transfer in the cleavage stage. Although many studies show that the success rate of transfer in the blastocyst stage is sometimes higher than the cleavage stage, such as, due to the lack of suitable endometrium, ET is needed in the cleavage stage (17-19).

In our study frequency of pregnancy was 41.2% and 22.7% in the blastocyst and cleavage transfer, respectively. Furthermore, the possibility of clinical pregnancy was 2.39 times higher in the blastocyst stage. Same as our study, it was shown that the possibility of clinical pregnancy is 1.3 times higher in the blastocyst group. In addition, no significant difference was observed between the groups in terms of pregnancy outcomes. It is suggested that the difference might be related to the development observed in recent years and the massive population of this systematic study. However, in line with the present study, the frequency of abortions and multiple births did not differ in the compared groups (15). In contrast with these studies, a study shows no significant difference in the pregnancy rate in the mentioned stages; however, their findings of the frequency of abortions were similar to the current study. It appears that the results of older investigations might be different from the new ones, which might be due to the difference in their methods of freezing, transferring, and storing the fetus (20).

Another study found that the rate of live birth in every IVF cycle was significantly lower in embryos transferred in the cleavage stage compared to the blastocyst stage (31.3% compared to 37.8%). Moreover, the number of embryos required for the first live birth was lower in the blastocyst stage (21). In the current investigation, although the number of transferred embryos were same in both groups, the clinical pregnancy rate was higher in the blastocyst group. Similar to these studies, Papanikolaou and colleagues showed that pregnancy and birth rate were higher in the blastocyst transfer rather than cleavage, when assessing 351 women under the age of 36. Their clinical pregnancy rates were 38.7% and 26.1% in the blastocyst and cleavage groups, respectively. In addition, their study suggested that due to the low rate of multiple pregnancies, transfer in the blastocyst stage can reduce maternal complications (22).

A study on infertile women under 35 yr declared that the implantation rate in blastocyst and cleavage stages was 40.16% and 11.43%, respectively. In line with the present study, they represented that the clinical pregnancy rate was higher in the blastocyst stage (62% vs. 29.7%). Therefore, their research showed that blastocyst transfer requires less embryo and can also increase the possibility of implantation and pregnancy (23). In another study it was found that the clinical pregnancy rate was 55.6% and 68.8%, using blastocyst and cleavage transfer, which was consistent with the present study (24).

In a systematic review it was revealed that the rate of live birth with blastocyst transfer was 1.39 times higher than cleavage. Its rate of clinical pregnancy was 1.27 times higher as well. In this study, the cancellation rate in women with blastocyst transfer was 2.21 times higher than women with cleavage transfer (25). The high rate of cancellation rate in the blastocyst stage is one of the major concerns when determining the treatment option for the women. Nevertheless, some claims that in women under the age of 36 who have undergone one or 2 stages of IVF, termination of blastocyst transfer is rare. It should be noted that further prospective studies are required in older women or women with recurrent implantation failures (22).

An investigation in 2017, examined 1627 ET cycles and showed that implantation rates in the cleavage and blastocyst stages were 48.98% and 60.68%, respectively. In contrast with the present study and some other studies, the rate of clinical pregnancy was not significantly different between the 2 groups in their research (67.5% in the cleavage stage vs. 71.5% in the blastocyst stage), which might have happened due to the close frequency of clinical pregnancies in both groups. Nonetheless, their other findings, including no significant difference between the 2 groups in terms of multiple births, preterm delivery, and low birth weight, were in line with other studies (26).

Even though the rate of clinical pregnancy in the blastocyst stage was higher in most studies, even those which did not find a significant difference between the 2 groups, it seems that the right time for ET is still controversial. However, the findings of this research suggest that transfer in the blastocyst stage is associated with a higher possibility of pregnancy and has no more risks than a transfer in the cleavage stage. Therefore, it appears that blastocyst transfer increases the possibility of clinical pregnancies. Still, its consequences, such as abortion, multiple births, preterm delivery, etc. are not different from the cleavage stage. Evaluating all possible pregnancy outcomes as well as the appropriate statistical population increased the validity of this study. However, the lack of assessment of embryonic factors such as live birth rate can be its main limitation.

## 5. Conclusion

In general, considering that the probability of clinical pregnancy in the blastocyst stage was significantly higher than the cleavage stage and no statistically significant differences were observed between the 2 stages of pregnancy, it can be concluded that the transfer in the blastocyst stage leads to better results.

##  Conflict of Interest

The authors declare that there is no conflict of interest.
